# Perfectionism, impostor phenomenon, and mental health in medicine: a literature review

**DOI:** 10.5116/ijme.5f54.c8f8

**Published:** 2020-09-28

**Authors:** Mary Thomas, Silvia Bigatti

**Affiliations:** 1 Department of Social and Behavioral Sciences, Indiana University Fairbanks School of Public Health at IUPUI, USA

**Keywords:** Perfectionism, impostor phenomenon, mental health, professional identity formation, medical culture

## Abstract

**Objectives:**

The aims of this review, focused on medical students, residents, and physicians, were a) to determine the levels of perfectionism and prevalence of impostor phenomenon, b) to assess the relationship between perfectionism, impostor phenomenon, and mental health, and c) explore how medical culture may influence these personality characteristics.

**Methods:**

A narrative literature review was conducted. Search terms were entered into PubMed, PsychINFO, Web of Science, EMBASE, and Google Scholar without date or geographic restrictions. The McMaster Critical Review Forms for Quantitative and Qualitative Studies were used for article appraisal. Final decisions on inclusion and exclusion were reached through discussion. Sixteen articles were included in this review and summarized in a data extraction table.

**Results:**

Medical students had similar perfectionism scores to other student groups but scored lower in maladaptive perfectionism. The overall prevalence of the impostor phenomenon ranged from 22.5% to 46.6%. More females (41% - 52%) experienced clinical levels of impostor phenomenon compared to males (23.7% - 48%). Most studies did not find an association between the impostor phenomenon and academic year of training. Both personality characteristics were associated with negative mental health effects. Medical culture can train for and/or exacerbate these characteristics, affecting professional identity formation. Both characteristics contribute to distress for learners during commonly-used teaching methods in medical education.

**Conclusions:**

Comprehensive changes in medical education that consider the relationship between medical culture, professional identity formation, impostor phenomenon, and perfectionism are needed. Longitudinal studies will help identify the implications of these findings for professional identity formation and medical education.

## Introduction

Accumulating evidence shows the risk of poor mental health in medical students and professionals. A recent global meta-analysis reported the overall prevalence of depression or depressive symptoms and suicidal ideation among medical students to be 27.2% and 11.1%, respectively.^1^ Of the students that were experiencing depression, only 15.7% sought psychiatric consultation.^1^ In residents, the prevalence of depression or depressive symptoms is estimated to be 28.8% worldwide.^2^ Within the United States, depression prevalence is estimated to be 58.2% for medical students, 50.8% for residents/fellows, and 40% for physicians.^3^

Studies have also been conducted to determine the prevalence rates of Burnout Syndrome, which is characterized by emotional exhaustion, depersonalization, and reduced personal accomplishment.^4^ Prevalence of burnout among medical students, residents, and physicians within the United States was 55.9%, 60.3%, and 51.4%, respectively in 2014.^3^ Relative to the general United States population, medical students, residents, and physicians are more likely to report burnout.^3^ Furthermore, the suicide risk for female physicians is 2.27 times that of the general female population, and for male physicians, it is 1.41 times higher than the general male population.^5^

Henning and colleagues^6^ conducted a study of psychological adjustment among multiple health professions students and determined that perfectionism and impostor phenomenon (IP) were the strongest predictors of medical students' psychological distress.

Henning suggested that future studies explore these personality characteristics, the role they might play in professional impairment, and how academic institutions may exacerbate them.^6^

### Perfectionism

Perfectionism is considered to be a multidimensional personality construct and has many definitions throughout the literature.^7^^,^^8^ Freud believed that perfectionism resulted from one's superego demanding superior achievement and behavior.^8^ Hewitt and Flett,^9^ who developed the Multidimensional Perfectionism Scale (HF-MPS), described perfectionism as a set of characteristics whereby the perfectionist sets and attempts to achieve unrealistic standards, focuses on and overgeneralizes failures, carries out stringent self-evaluation, and engages in an all-or-nothing mentality that classifies each outcome as either a complete success or complete failure. Their scale measures three dimensions of perfectionism: self-oriented perfectionism, other-oriented perfectionism, and socially-prescribed perfectionism.^9^ Self-oriented perfectionism involves the set of characteristics previously described including setting unrealistic goals for oneself and engaging in stringent self-evaluation.^9^ Other-oriented perfectionism involves setting unrealistic expectations for and critically evaluating others.^9^ Socially-prescribed perfectionism reflects the need to meet expectations set by others in order to gain their approval.^9^ In socially-prescribed perfectionism the perceived locus of control (the degree to which one believes he or she controls a situation and its outcome) is external or controlled by others.^9^ Since its introduction, the HF-MPS has been used in several studies to further explore these dimensions.^6^^,^^7^^,^^10^ Another frequently used perfectionism scale was developed by Frost and associates. The Frost Multidimensional Perfectionism Scale (F-MPS) includes six components of perfectionism: concern over mistakes, personal standards, doubts about actions, organization, parental expectations, and parental criticism.^11^

Perfectionism can be either adaptive (positive) or maladaptive (negative).^11^^, ^^12^ Neither the HF-MPS nor the F-MPS directly measure these subtypes; therefore, they are generally measured using their respective associated subscales.^7^^,^^10^^, ^^13^^-^^15^ Adaptive perfectionism is driven by a desire for success and goal attainment.^12^ It involves a high level of organization and personal standards, conscientiousness, and self-oriented perfectionism.^11^ Maladaptive perfectionism is driven by a fear of failure and results in the need to conceal imperfections about oneself.^12^ It has been associated with socially-prescribed perfectionism, concern over mistakes, and doubts about actions.^11^ The main difference between the two subtypes is that adaptive perfectionists derive satisfaction from their efforts because they are flexible enough to allow for occasional mistakes - a trait not seen in maladaptive perfectionists.^12^ Maladaptive perfectionism and its components have been associated with a multitude of detrimental health effects including but not limited to anxiety,^16^^,^^17^ depression,^11^^,^^18^^,^^19^ bulimia nervosa,^20^ anorexia nervosa,^21^^,^^22^ chronic fatigue syndrome,^13^^,^^23^^,^^24^ and lower levels of engagement in preventive health measures.^25^

### Impostor phenomenon

The impostor phenomenon was first described by Dr. Pauline Clance as an experience whereby individuals feel that they do not deserve their successes despite objective evidence to the contrary; therefore, they feel they will eventually be exposed as an impostor.^26^ Individuals who suffer from IP do not internalize their success.^26^ For individuals with IP, a task leads to the development of anxiety, which causes one to either over-prepare or procrastinate and then rush to prepare shortly before the deadline.^27^ If one over-prepares, the belief becomes that one must work harder than others to do well, and for that reason, he or she is an impostor.^27^  If one procrastinates, the belief becomes that one was able to fool others once again by rushed preparation at the last minute, and for that reason, he or she is an impostor.^27^ In both situations, the individual discounts any positive feedback received and does not internalize the success.^27^ The individual begins to feel as though dread and worry are necessary for success and attributes this success to an external source – luck.^27^ The sense of accomplishment and relief is short-lived as the next challenge starts the cycle over again.^27^ 

Clance identified six characteristics that may be present in individuals suffering from IP: (1) the impostor cycle (2) the need to be the best (3) superman/woman aspect (4) fear of failure (5) denial of one's competence and (6) fear of success.^28^ Many of these are interrelated. People experiencing IP feel the need to be the best.^28^ This need causes them to set high and unattainable standards for themselves, which leads to the development of the superman/woman aspect of the syndrome.^28^ The anxiety that is derived from an achievement-related task is the result of a fear of failure, which leads to feelings of shame and humiliation.^28^ They internalize failure but attribute success to external factors. They contrive reasons as to why they do not deserve praise or credit.^28^ They fear success because they worry that their success will generate higher expectations from others that they will inevitably not be able to meet.^28^ Not surprisingly, IP has been associated with negative mental health effects including but not limited to anxiety,^29^^,^^30^ depression,^30^ neuroticism,^31^ and low self-esteem.^30^^,^^32^

### The relationship between perfectionism and impostor phenomenon

Throughout the literature, characteristics of perfectionism are mentioned in studies assessing IP. Setting and attempting to attain unrealistic goals for oneself is characteristic of both IP and self-oriented perfectionism.^28^^,^^32^ People experiencing IP may have similar cognitive distortions as those seen in perfectionism, where the individual will engage in an all-or-nothing mentality and overgeneralize mistakes.^29^ Self-evaluative perfectionism aspects including but not limited to concern over mistakes, need for approval, and rumination were found to be positively and significantly associated with Clance's Impostor Scale (CIPS) scores.^33^ Thompson and colleagues^29^ also found that the Concern over Mistakes subscale of the F-MPS positively and significantly correlated with CIPS scores.

To date no literature review assessing IP, perfectionism, and mental health in medical students and professionals has been conducted. A comprehensive understanding of the relationship between these variables and medical culture is needed to guide medical education decision-making and to improve the mental health of medical students and professionals. Given the prevalence of depression and burnout among these populations and the findings of the Henning study, this literature review has three objectives (1) determine the levels of perfectionism and prevalence of IP in medical students, residents, and physicians (2) assess the relationship between perfectionism, IP, and mental health in medical students, residents, and physicians and (3) explore how medical culture influences these personality characteristics.

## Methods

### Study design

A narrative review of the literature was conducted, including the use of search terms, eligibility criteria, and article quality assessment with the purpose of summarizing available information and identifying gaps in this area of research. Ethical approval was not necessary as this review did not require the collection of primary data.

### Search strategy

In order to find relevant articles to include in this review, the search terms below were entered into PubMED, PsychINFO, Web of Science, EMBASE, and Google Scholar.

(Perfectionism OR perfectionist) AND ("medical students" OR residents OR physicians OR doctors), (Impostorism OR imposterism OR "impostor syndrome" OR "imposter syndrome" OR "impostor phenomenon" OR "imposter phenomenon") AND ("medical students" OR residents OR physicians OR doctors).

No date or geographic restrictions were placed on these searches.

### Eligibility criteria

In general, only full text, scholarly journal articles that were available in English were eligible for inclusion in this review. Qualitative and quantitative studies were eligible. Table 1 lists the inclusion and exclusion criteria for this review.

### Article analysis 

The quality of each article was assessed by the first author using the McMaster Critical Review Form for Quantitative Studies and the McMaster Critical Review Form for Qualitative Studies as guides for important areas to assess in research articles.^34^^,^^35^ The McMaster Critical Review Form for Quantitative Studies assesses the following components of each study: study purpose, literature review, sample characteristics and size, reliability and validity of outcome measures, statistical analysis of results, and study limitations.^34^ The McMaster Critical Review Form for Qualitative Studies assesses the following components of each study: aims of the research, methodology, research design, recruitment strategy, data collection method, the relationship between the researchers and participants, and ethical considerations.^35^ Based on these components, papers with concerning flaws (i.e. issues with statistical analysis, conclusions not supported by the presented data, lack of discussion on study results and limitations) were not included in this review. When author one was uncertain about a study's quality, author two was consulted for final decisions on inclusion and exclusion. A final consensus was reached through discussion.

**Table 1:** Inclusion and exclusion criteria

Inclusion Criteria	Exclusion Criteria
Scholarly journal article	Combines data for one of the listed population groups with another population group that is not listed
Written in English	No validated survey instrument utilized
Full text available	Use of a proxy to measure impostor phenomenon or perfectionism
Quantitatively assesses or qualitatively explores impostor phenomenon and/or perfectionism	
Population must include medical students, medical residents, and/or physicians	
Must correlate impostor phenomenon or perfectionism with a mental health-related topic (if the study attempts to make a correlation)	

### Data extraction and synthesis

Articles that met the inclusion and exclusion criteria were included in this review. Studies are summarized in a data extraction table in Appendix A – Studies Selected for Review. This table was constructed by the authors and includes author name(s), date of study, origin of study, methodology, participant characteristics, survey instrument utilized, and major findings of each study. Included articles were first sorted by study type – quantitative or qualitative. Quantitative studies were then sorted by study subject - perfectionism, IP, or both. Common categories among articles within each group were identified. Final categories within the perfectionism group included perfectionism scores and perfectionism and mental health. Final categories within the IP group included IP scores and prevalence, IP and gender, IP and academic year, and IP and mental health. Findings from the limited amount of qualitative studies were summarized and not categorized further.

## Results

### Study characteristics

**Figure 1:**
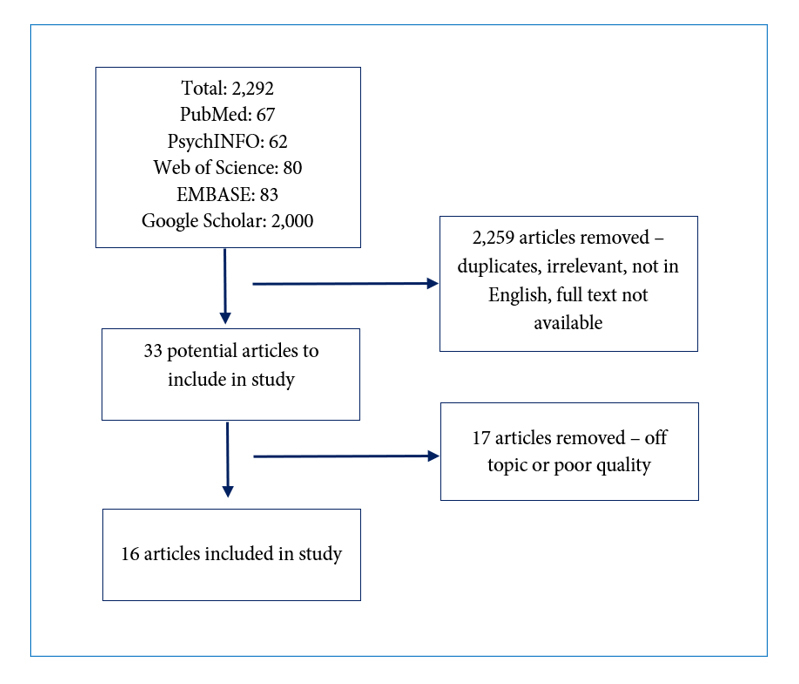
Article selection process

A total of 2,292 articles were retrieved through database searches. PubMED returned 67, PsychINFO returned 62, Web of Science returned 80, and EMBASE returned 83. Although Google Scholar initially returned 38,700 and 2,030 results after entering search terms one and two, respectively, only the first 1,000 results for each search were available to view. Therefore, a total of 2,000 results were recorded for the Google Scholar search. Upon title and abstract review, 2,259 articles were removed because they were duplicates, irrelevant to the study topic, not in English, and/or no full text version was available. Thirty-three articles remained. After a full text review of each of the remaining articles,17 were removed either because they were off topic or of poor quality; therefore, a total of 16 articles were included in this review. This process is detailed in Figure 1.

Seven articles explored perfectionism, eight articles explored IP and one article explored both topics. Thirteen articles were quantitative and three articles were qualitative. Fifteen articles were cross-sectional and one article was longitudinal. Five of the studies were conducted within the United States, three in Canada, and one in each of the following countries – United Kingdom, Romania, Saudi Arabia, Nigeria, Malaysia, India, Germany, and Korea.

### Perfectionism scores

The majority of the studies on perfectionism assessed levels of perfectionism in medical students. Studies that used the HF-MPS assessed self-oriented, socially-prescribed, and other-oriented perfectionism.^10^  Perfectionism scores were highest in the self-oriented category (50.2-69.9) and lowest in the socially-prescribed category (38.71-49.1).^6^^,^^7^^,^^10^ These studies did not compare the results with normative data, which has a mean self-oriented perfectionism score of 65.91 and a mean socially-prescribed perfectionism score of 54.75 for student populations.^36^ While Enns and colleagues^7^ and Henning and colleagues^6^  found similar scores among medical students in Canada and the United States, Seeliger and colleagues^10^ reported much lower scores for medical students in Germany. Enns and colleagues^7^ also found that compared to a random sample of art undergraduate students, medical students had a higher mean personal standards score (25.8 vs. 23.4; p < 0.01), lower doubts about actions (9.6 vs. 11.5; p < 0.01) and lower maladaptive perfectionism mean scores (-0.786 vs. 0.263; p<0.05). No statistically significant differences for adaptive perfectionism were found between the art undergraduate and medical students.^7^ Henning and colleagues^6^ reported similar findings in that medical students did not report statistically significant differences in perfectionism levels from undergraduate, dental, nursing, and pharmacy students.

The remaining study that explored perfectionism levels used a different method and scoring system that cannot be compared to the studies mentioned above. Aboalshamat and colleagues^37^ used the Perfectionist Self-Presentation Scale, which assesses perfectionistic self-promotion (the individual proclaims and displays perfection), non-display of imperfection (the individual avoids behavioral demonstrations of imperfection), and nondisclosure of imperfection (the individual avoids admitting to imperfection) to determine levels of perfectionism in medical students in Saudi Arabia. Students' mean scores for self-promotion, non-display of imperfection, and nondisclosure of imperfection were 42.68, 41.49, and 29.32, respectively.^37^ These scores were not statistically significantly different from dentistry students.^37^

### Perfectionism and mental health

Many of the studies above also assessed the association of perfectionism with various aspects of mental health.^6^^,^^7^^,^^10^^, ^^38^^,^^39^ Because the components and categories of perfectionism assessed differed by study, results will be discussed by perfectionism component. Enns and colleagues^7^ conducted a longitudinal study of medical students in Canada. At baseline, maladaptive perfectionism was positively correlated with depression (p<0.001), hopelessness (p < 0.001), suicidal ideation (p<0.001), neuroticism (p<0.001), and one’s view of what constitutes an acceptable level of performance (p< 0.01).^7^ Adaptive perfectionism was positively correlated with neuroticism (p<0.01), conscientiousness (p<0.001), one’s view of what constitutes an acceptable level of performance (p<0.001), and one’s belief in his or her ability to achieve (p <0.001).^7^ A regression analysis determined that maladaptive perfectionism, measured at time one, correlated with depression (p= 0.03) and hopelessness (p=0.05) but not suicidal ideation at time two (six months later).^7^ These findings were statistically significant after controlling for age, gender, and medical school year.^7^

In support of these findings, Seeliger and colleagues^10^ found that the strongest predictor of the occurrence of depression and anxiety in medical students in Germany was maladaptive perfectionism. The statistically significant (p< 0.01) correlations found for the HF-MPS dimension of socially-prescribed perfectionism and mental health in medical students in Korea were as follows: academic burnout (r = 0.428), psychological distress (r = 0.38), cynicism (r = 0.349), emotional exhaustion (r = 0.353), and self-confidence (r=-0.374).^38^

In support of these findings, Henning and colleagues^6^ found that socially-prescribed perfectionism was one of the greatest predictors of psychological distress in medical students in the United States after controlling for sex, marital status, and prior mental health treatment.

Craiovan^39^ used Hill and colleagues’^40^ Perfectionism Inventory comprised of eight constructs including concern over mistakes, high standards for others, need for approval, organization, parental pressure, planfulness, rumination, and striving for excellence in order to assess conscientious and self-evaluative perfectionism. The statistically significant (p<0.01) correlations Craiovan^39^ found in physicians in Romania were as follows: perceived stress level (r=0.49), emotional tiredness (r=0.67), depersonalization (r=0.73), personal accomplishment (r=0.65), obsession (r=0.33), psychoticism (r=-0.59), paranoia (r=0.32), sensitivity (r= 0.42), somatization (r=0.56). Burnout, measured via three subscales, including emotional tiredness, personal accomplishment, and depersonalization using the Maslach Burnout Inventory, was positively associated with perfectionism.^39^ In support of these findings, Yu and colleagues^38^ found a positive correlation (p<0.01) between socially-prescribed perfectionism and academic burnout in medical students. However, Aboalshamat and colleagues^37^ found no association between work burnout and perfectionism in medical students.

### Impostor phenomenon scores and prevalence

Seven of the eight studies utilized CIPS^6^^,^^41^^-^^46^ and the remaining study utilized the Young Impostor Scale, an eight-item instrument that assesses impostor-like feelings.^47^ Responding positively to five of the eight questions is considered a positive finding for IP.^47^ CIPS determines whether individuals experience IP characteristics and, if so, to what extent by quantifying responses to several questions on a Likert scale from 1 (not at all true) to 5 (very true). The scoring yields four categories of IP: low (≤ 40), moderate (41-60), high (61-80), and intense (>80).^28^ Two of the seven studies reported findings using these cutoffs.  The other five studies used a score of 62 as a cutoff score^6^^,^^42^^-^^44^^,^^46^ as suggested in Holmes and colleagues.^48^

Using the cutoffs suggested by CIPS, one study of medical students in Nigeria found that 22.5% of participants experienced high levels of IP^41^ (>60) and a study including medical interns in India found that 46.6% of participants experienced high (>60) or intense (>80) levels of IP.^45^ Three studies that used a cutoff score of 62 and provided an overall IP percentage found that prevalence ranged from 33% to 45.7% for medical students and residents.^42^^,^^44^^,^^46^ Therefore, overall IP prevalence ranged from 22.5% to 46.6% for medical students and residents.^42^^,^^44^^,^^46^ Furthermore, mean impostor scores ranged from 46.97 to 61.2 in medical students, interns,residents, and surgeons, with medical students in Nigeria having the lowest mean score and residents in Canada having the highest mean score.^6^^, ^^41^^,^^43^^-^^45^

### Impostor phenomenon and gender

Most (six of eight) studies reported results based on gender.^42^^-^^47^ Mean CIPS scores for female medical students and residents ranged from 46.14 – 65.2^41^^,^^42^^, ^^44^^, ^^46^ with the highest scores reported in Canada (65.2)^44^ and Malaysia (60.3).^42^ Mean CIPS scores for male medical students and residents ranged from 47.4 – 62.4^41^^,^^42^^,^^44^^,^^46^ with the highest scores reported in Malaysia (62.4)^42^ and Canada (56.4).^44^Table 2  shows the percent of males and females in each study that reported clinical levels of IP. All study results are based on a cut off value of 62 to determine clinical IP except for the results from India, which reported percentages based on the four CIPS cutoffs. The results for medical students in the United States used the Young Impostor Scale. The medical internship year in India is the final year of the medical education curriculum and is similar to postgraduate training in many places outside of Asia.^49^ Due to differences in terminology among medical education systems, "intern" was considered to be its own category.

As shown in Table 2, except for the study conducted in Malaysia, a higher percentage of females compared to males experience clinical IP regardless of country or level of training. Three of the six studies found a statistically significant difference between female and male IP percentages and/or scores.^44^^,^^46^^,^^47^ Villwock and colleagues^47^ found that female gender was significantly associated with IP (p = 0.004). Oriel and colleagues^46^ found statistically significant differences between female and male mean IP scores (p = 0.03) and IP percentages (p= 0.02). Likewise, Legassie and colleagues,^44^ found statistically significant differences between female and male mean IP scores (p = 0.03).

**Table 2:** Impostor phenomenon by level of training, country, and gender

Level of Training	Country of Study	Females	Males
Medical students	Malaysia	44.23%	48%
Medical students	United States^*^	49.4%	23.7%
Residents	United States^*^	41%	24%
Residents	Canada^*^	52%	31.8%
Residents and physicians	United States	44%	36%
Interns	India	49%	42%

### Impostor phenomenon and academic year of training

Four studies assessed for a relationship between IP and academic year of training.^43^^,^^44^^,^^46^^,^^47^ Legassie and colleagues^44^ Leach and colleagues,^43^  and Oriel and colleagues^46^ found no statistically significant association between IP symptoms and year of training in residents and physicians. Although, Leach and colleagues^43^ found that general surgery residents had a significantly higher mean CIPS score than surgeons (61 vs 51.1; p = 0.017). However, Villwock and colleagues^47^ found that IP levels peaked in the fourth year of training for medical students (p = 0.015).

### Impostor phenomenon and mental health

All eight of the quantitative studies found statistically significant associations between IP and at least one aspect of mental health, including self-esteem, burnout, depression, anxiety, and psychological distress.^6^^,^^41^^-^^47^ Among medical students, Henning and colleagues^6^ found that the CIPS score was the greatest predictor of psychological distress and that psychological distress was negatively correlated with the academic year (p<0.01). Henning and colleagues.^6^ did not report the correlation between CIPS scores and academic year, however.6 Four of the studies assessed for and found a statistically significant negative correlation between self-esteem and IP.^41^^,^^42^^,^^45^^,^^46^ This correlation was found for medical students in Nigeria (p = 0.001)^41^ and Malaysia (p<0.0001)^42^ interns in India (p<0.05)^45^ and residents in the United States (p< 0.0001).^46^

Three studies assessed for and found a statistically significant correlation between one or more subscales of burnout and IP.^43^^,^^44^^,^^47^ IP was found to be positively correlated (p< 0.05) with the depersonalization, emotional exhaustion, exhaustion, and cynicism components of burnout in medical students in the United States^47^ and negatively correlated with the personal accomplishment subscale of burnout in medical students in Canada.^44^ Villwock and colleagues^47^ determined medical students in the United States with IP experienced higher levels of exhaustion (77% vs 58%), cynicism (69% vs 29%), and emotional exhaustion (48% vs 26%) than those without IP.  In a study of residents and physicians in the United States, it was determined that the odds of developing symptoms of burnout were 3.95 times greater for residents and physicians with IP;^43^ 12 (60%) of the residents and physicians with IP reported symptoms of burnout compared to 8 (40%) of residents and physicians without IP.^43^ This study did not assess burnout components individually.

Two of the studies assessed for and found a correlation between anxiety, depression, and IP.^42^^,^^46^ Ikbaal and colleagues^42^ found statistically significant (p<0.0001) positive correlations between CIPS scores in medical students in Malaysia and anxiety and depressive symptoms. Likewise, Oriel and colleagues.^46^ found that in residents in the United States, IP scores significantly and positively correlated (p< 0.0001) with state anxiety, trait anxiety, and depressive symptoms. Both studies also determined that anxiety and depression were predictors of IP.^42^^, ^^46^ The study including medical students in Malaysia found that participants with IP were 5.16 times more likely than participants without IP to report feeling unprepared to face challenges during residency and 1.98 times more likely to feel like quitting their current medical course.^42^

### Qualitative findings related to perfectionism, IP, and medical culture

Two qualitative studies explored perfectionism. In the first, Cope and colleagues^50^ interviewed and observed surgeons and surgical residents in an academic hospital in the United Kingdom in order to further understand the development of professional identity using Bandura's social learning theory and transformational learning as frameworks for their study. Combined, these theories suggest that learners observe and mimic their teachers' behaviors causing changes in the learners' identities.^50^ Therefore, researchers in this study theorized that medical residents become immersed and socialized into the surgical culture and that this immersion is involved in identity formation within the medical setting.^50^ Through interviews, perfectionism was identified as an important attribute of a surgeon and participants described learning to be a perfectionist.^50^ In particular, they learned that minor errors should be avoided and precision and faultlessness are to be valued.^50^

In the second study, Bynum and colleagues^51^ interviewed Internal Medicine residents in the United States to explore triggers of shame and factors that explain why these triggers cause shame. Triggers of shame related to learning included inadequate test scores, providing incorrect answers during rounds or noon report, being "pimped" (a potentially anxiety-provoking process whereby learners are asked a series of questions by a supervisor, often in public, to determine the extent of the learner's knowledge),^52^ morbidity and mortality conferences, and receiving negative feedback.^51^ Residents identified perfectionism as a contributory factor.^51^ They described being very critical of themselves and feeling deficient when they were unable to meet unobtainable goals.^51^ Residents also mentioned viewing themselves as inadequate despite objective evidence to the contrary, suggesting that IP may also contribute to shame.^51^

IP was explored by LaDonna and colleagues^53^ who interviewed physicians in Canada about their experiences with underperformance and self-assessment.^53^ Data collected early in the study identified IP as a feature of some of the physicians' experiences.^53^ IP was perceived to occur at the extreme end of self-doubt, which was reported to be a recurrent feeling that is triggered or exacerbated by transition periods in training.^53^ The impact of feedback on feelings of self-doubt were mixed.^53^ Some participants believed that feedback was reserved for underperforming learners and therefore, no feedback meant they were performing well; however, others reported that a lack of feedback exacerbated self-doubt.^53^ For participants who identified as impostors, positive performance feedback was not enough to negate the self-doubt.^53^ The authors postulate that IP and self-doubt act as a barrier to receiving feedback and create psychological distress.^53^ Physicians reported that poor patient outcomes, patient complaints, and negative teaching evaluations contributed to self-doubt after residency.^53^

Participants mentioned that the culture of medicine could perpetuate and cause feelings of insecurity, making it difficult to manage feelings of self-doubt.^53^ Showmanship was widely endorsed as a cultural value in medicine and medicine was viewed by some to be a performance art.^53^ Participants discussed how patients perceive the white coat as a symbol of physician competence; however, this cultural norm also exacerbated IP symptoms.^53^ Acting the part of a competent physician was used as a coping mechanism to hide feelings of self-doubt.^53^ Despite these coping mechanisms, persistent feelings of inadequacy were attributed, in part, to the notion that discussing these feelings and IP is taboo in the culture of medicine.^53^ Participants felt that admitting to or seeking help for such feelings is perceived as an admission of weakness in medicine.^53^

## Discussion

Perfectionism and IP have been found in medical students, interns, residents, and physicians. Perfectionism is considered to be a multidimensional personality construct with adaptive and maladaptive subtypes.^11^^,^^12^ Three recognized dimensions are self-oriented, other-oriented, and socially-prescribed perfectionism.^9^ Individuals with IP believe their successes are the result of external factors and fear they will be discovered as impostors.^28^ Several similarities exist between the two characteristics, including setting unrealistic goals, all-or-nothing mentality, and self-evaluative perfectionism.^28^^,^^29^^,^^32^

### Prevalence

Research consistently showed that medical students have similar perfectionism scores to other student populations.^6^^,^^37^ When compared to other student groups, medical students had higher personal standards and lower maladaptive perfectionism scores, but were not significantly different in adaptive perfectionism scores.^7^ The IP prevalence rates reached 52% in female residents and 49% in female interns which are higher than the percentages seen in their male counterparts.^44^^,^^45^ Malaysia was the only country to report higher prevalence rates in males compared to females. The difference in IP scores by gender may be related to the gender makeup of each particular environment.^54^ Environments with less female role models may show higher IP scores in female participants. It could also reflect cultural views on the education of females and gender roles. IP may develop in response to internal conflict that results when high achieving women stray from traditional societal gender roles.^27^ Similar prevalence levels for medical students, interns, residents, and physicians show that IP occurs at every level of training and that having successfully completed medical training does not remove the IP. Similar levels of IP prevalence for residents in the fields of general surgery, internal medicine, and family medicine provides evidence of the transcendence of IP across medical specialty bounds.

Three of four cross-sectional studies showed no correlation between year of training or practice and IP in residents and physicians.^44^^,^^46^^,^^51^ Villwock and colleagues^47^ found that for medical students, higher levels of IP occurred in the fourth year of training. The higher IP levels in the fourth year may be triggered by the upcoming transition to residency training.^47^ The conflicting findings between Villwock and colleagues^47^ and other cross-sectional studies may indicate changes in these variables across the years, either related to generational characteristics of students or changes in medical training. Some physicians reported that transition periods seemed to exacerbate or trigger feelings of IP.^53^ These findings suggest the need to conduct longitudinal studies that assess these personality characteristics and their potential association with medical training. Longitudinal studies assessing changes in perfectionism and IP scores throughout the entirety of medical school training have not been explored among medical students, possibly because as personality characteristics they are considered stable.

### Relations to mental health

Both perfectionism and IP are associated with poorer mental health. Perfectionism (depending on the subtype) was found to be positively correlated with depression, anxiety, suicidal ideation, burnout, psychological distress, cynicism, and low levels of self-confidence, among other mental health aspects.^7^^,^^38^^,^^39^ Likewise, IP was found to be positively correlated with depression, anxiety, burnout, psychological distress, and low self-esteem.^39^^-^^45^ Several studies also provided results that supported that IP and perfectionism are among the strongest predictors of psychological distress in medical students and that perfectionism is a strong predictor for anxiety and depression.^6^^,^^7^^,^^38^ This review shows a significant overlap between IP and perfectionism in relation to mental health.

Of the studies included in this review, only one study assessing the relation between these personality characteristics and mental health was longitudinal. Enns and colleagues^7^ showed that while maladaptive perfectionism was associated with hopelessness, depression, and suicidal ideation at time one, it was not associated with suicidal ideation at time two, possibly suggesting that a lesser degree of psychological distress occurs as medical students progress in their training. However, the difference between time one and time two was only six months and therefore, it is not known how the relationship between perfectionism and mental health changes over the entirety of medical school training. Using a cross-sectional methodology including students at various levels of training, Henning and colleagues^6^ found that psychological distress decreased with academic year for medical students and that IP and socially-prescribed perfectionism were the strongest predictors of this distress.

Results were conflicting regarding the association between both personality characteristics and burnout.^37^^,^^38^^,^^44^^,^^51^ These differing conclusions may result from assessing different subtypes of perfectionism or the individual components of burnout as opposed to overall burnout scores. The 2019 Medscape National Physician Burnout, Depression, and Suicide report showed that 44% of physicians reported symptoms of burnout.^55^ Ten percent of physicians reported such severe burnout symptoms that they were considering leaving medicine.^55^ Furthermore, 14% of respondents endorsed thoughts of suicide, and 1% had attempted suicide.^55^ Burnout is an independent risk factor for reduced professional work effort and the intent to leave medicine, exacerbating the current physician shortage.^56^^,^^57^ Electronic medical records, loss of autonomy, and excessive clerical responsibilities are among the cited contributors to burnout.^56^ The results reported in this review suggest that IP and perfectionism may also be contributors.

### Relations to the medical culture

Qualitative studies provided insight into the relationship between the culture of medicine, perfectionism, and IP. Physicians indicated certain aspects of medical culture, such as valuing showmanship and symbolism of the white coat, could exacerbate IP and self-doubt.^53^ The stigma attached to seeking help for mental health issues makes it difficult to cope with such feelings.^53^

Cope and colleagues^50^ discussed the relationship between surgical culture and professional identity formation. Professional identity formation in medical students is the result of the convergence of multiple domains, including psychosocial identity development, professionalism, and formation^58^ and is related to both perfectionism and IP. The characteristics of a student's identity that are received positively by other students, professionals, and patients will remain intact in a student's professional identity and vice versa.^58^ Students who come to view themselves as accepted in their environment develop a healthy professional identity, which allows them to eventually assume the physician role without experiencing IP.^58^ Surgical residents constructed an identity that valued perfection after becoming immersed and socialized into the surgical culture.^50^ The faculty members in this study also endorsed this personality change.^50^ These findings suggest that certain individual characteristics may be learned once immersed within a culture as opposed to individual characteristics attracting medical students to certain medical specialties.^50^

Professional identity formation is also influenced by interactions with family and friends.^59^ Internal distress can occur when family and friends expect medical students to have answers to clinical questions even in their preclinical years, contributing to the development of IP and negatively impacting professional identity formation.^59^ In turn, many medical students often feel as though they only have a superficial grasp of medical knowledge while believing that their fellow classmates have a more profound understanding of the material. Such internal distress may begin as early as the first year of medical school.^59^ This belief that one's peers are higher achieving is also reported by internal medicine residents.^51^

This literature review is the first to summarize available data on IP and perfectionism in medical students, residents, and physicians and to explore these topics globally. Similar results across multiple countries provide evidence that IP and perfectionism in medicine are global issues. Although this area of research has not been extensively studied, it is clear that medical students, residents, and physicians in multiple specialties and across multiple cultures are at risk of poor mental health and even suicide and that these problems are associated with both perfectionism and IP.

### Implications for medical education

Although IP and perfectionism are considered personality characteristics, qualitative studies suggest that certain aspects of medical education can contribute to their development and/or exacerbation.^50^^,^^51^^,^^53^ Transition periods are a key focus area when discussing changes to medical education as they can exacerbate IP.^53^ This review showed that IP occurs during and after training and within multiple specialties. It is important to consider that transition periods still occur for practicing physicians, highlighting the need for continued guidance and mentorship after training.

Perfectionism and possibly IP were identified as contributory factors in the development of shame during grand rounds, noon report, morbidity and mortality conferences, receiving feedback, and being "pimped."^51^ These situations provide opportunities to enlighten trainees on IP and perfectionism and collectively explore ways to teach in such an environment without causing shame for learners. Globally, these findings highlight the need for changes in medical education in addition to reducing the stigma of seeking mental health care for people with these personality characteristics.

IP and perfectionism add an additional layer of complexity to providing and receiving feedback.^51^ People with IP assess themselves using inaccurate frames of reference^51^ and do not internalize success,^28^ thus preventing effective feedback.^51^ Differing interpretations of receiving feedback by study participants indicate the need for feedback providers to indicate to the recipient how they use and provide feedback as a tool for improvement.

Within the medical culture, each subspecialty serves as a subculture that medical students must learn to navigate.^59^ Mentors play an integral part in this process as medical students construct their professional identities.^59^^,^^60^ During this exploratory process, students internalize the bioethical principles of the profession and shape their morals through social learning and clinical experiences.^58^ Given Bandura's social learning theory, it is postulated that earlier clinical experiences may help professional identity formation.^58^ In order to make effective, comprehensive changes in medical education, the relationship between medical culture, professional identity formation, IP, and perfectionism must be considered.

### Study limitations

There are several limitations to this study. Article quality assessment was primarily performed by one author. Only English-language articles were included in this review and results are based on a limited number of studies. Some of the studies that were included in this review used small sample sizes which reduces the power and external validity of the results of those studies. Differences in survey instruments and a lack of normative data for some instruments make a comparison between groups and among studies difficult. While some studies attempted to determine the predictors of poor mental health, causation cannot be shown with the available studies. The purpose of qualitative research is to gain a deeper understanding of a certain topic and is not necessarily to generate generalizable data. Therefore, the information from the qualitative studies discussed in this report is not necessarily generalizable to other populations. Finally, several of the studies reviewed may no longer reflect the current experiences of medical students. Medical training has undergone significant change since many of these studies were published,^61^ and it is not known whether and how much these changes have impacted medical culture, perfectionism, or IP.

### Recommendations for future research

Research on perfectionism and IP would be facilitated by the development of normative data for IP and categorization of perfectionism levels using cut off values. Normative data for IP would allow for comparisons with the general population. More studies assessing perfectionism and IP are needed, particularly in residents and physicians in various specialties to determine prevalence rates within each population and across specialty bounds. Furthermore, having more data on perfectionism and burnout would help disentangle the currently conflicting results.

Longitudinal studies on these populations are scarce. This type of study design can better assess how IP and perfectionism might differ with the level of training. Differentiating perfectionism subtypes may better explain the experience of perfectionism in medical personnel, from training through practice. Cope and colleagues^50^ suggested that surgical residents learn perfectionism through socialization into the surgical culture. A longitudinal study design following medical students into multiple medical specialties would elucidate the relationship between training and these personality characteristics and determine if perfectionism is learned through training in other medical specialties. Finally, longitudinal studies, including premedical students, are needed to further elucidate the relationship between IP, perfectionism, professional identity formation, mental health, and medical culture.

Efforts to adapt the curriculum to newer generations and improve professional identity development,^62^ to design foundational courses that facilitate learning and can be used across medical schools,^63^ and to design curricula to improve wellness^64^ are just some examples of potential positive changes to medical training that have either occurred or may occur in the near future. More research is needed to determine additional areas of focus in medical education that may contribute to IP and perfectionism. Research that explores the current experience of medical students may provide better solutions to the problems identified in this review.

## Conclusions

Despite identifying the correlations among perfectionism, IP, and mental health, these characteristics remain understudied among medical personnel, which is evident by the number of studies included in this review. While there are stressors that are inherent to the medical field, certain aspects of the culture that promote these problems can be mitigated or eliminated.^65^ The studies included in this review give insight into the aspects that may specifically influence perfectionism and IP. More research is needed in order to design and implement a multifaceted solution to this issue.

### Acknowledgements

The authors would like to thank Dr. Carole Kacius for support and encouragement while conducting this literature review.

### Conflict of Interest

The authors declare they have no conflict of interest.

## Supplementary material

Supplementary file 1 Appendix A. Studies Selected for Review
