# Supplementary material for: Perfectionism, impostor phenomenon, and mental health in medicine: a literature review
Source: Int J Med Educ. 2020 Sep 28;11:201–13. doi: 10.5116/ijme.5f54.c8f8 (PMC7882132; doi:10.5116/ijme.5f54.c8f8)
Supplement: Supplementary file 1 — Appendix A. Studies Selected for Review [file ijme-11-201-S1.pdf]

Appendix A

Studies Selected for Review

Authors	Date	Origin of study	Methodology	Participants	Survey instrument	Major findings
Aboalshamat, K., et al.	2017	Saudi Arabia	Quantitative, Cross sectional; Hard-copy and electronic surveys in English and some terms in Arabic	282 medical students	CBI, Perfectionist Self-Presentation Scale	High level of burnout among medical students; Levels of perfectionism in medical students were comparable to dentistry students; No significant correlation between perfectionism and burnout in medical students
Bynum, W., et al.	2019	United States	Qualitative Hermeneutic; Individual, semi-structured interviews	12 internal medicine residents	N/A	Perfectionism contributed to shame for many participants
Cope, A., et al.	2017	United Kingdom	Qualitative, Grounded theory, Individual, semi-structured interviews, ethnographic observation followed by further descriptive interviews	16 postgraduate surgeons	N/A	Many participants described learning how to be a perfectionist, which suggests perfectionism is considered core to the professional identify construction of a surgeon
Craiovan, P.	2014	Romania	Quantitative, Cross sectional; Questionnaires	32 physicians	Perfectionism Inventory, Perceived stress scale, MBI, and Symptom Checklist	Strong to moderate positive association between perfectionism, burnout symptoms, & certain psychopathological symptoms in doctors (anxiety and depression)
Egwurugwu, J.N., et al.	2018	Nigeria	Quantitative, Cross-sectional descriptive; Questionnaires	200 medical students	RSES, CIPS	Less than half of medical students displayed at least moderate levels of IP; Negative and significant correlation between IP and self-esteem
Enns, M., et al.	2001	Canada	Quantitative, Longitudinal; Mailed questionnaires	96 medical students	MPS – Hewitt MPS - Frost; NEO Five-Factor Inventory (N and C scales), Beck Depression Inventory, Beck Hopelessness Scale, Suicidal Ideation Questionnaire, Self-report academic measures	Maladaptive perfectionism was significantly and positively correlated with depression, hopelessness, suicidal ideation, and neuroticism; Maladaptive and adaptive perfectionism were significantly and positively correlated with dissatisfaction with academic performance
Henning,K., et al.	1998	United States	Quantitative, Cross sectional, Questionnaires	477 health professions students, 221 medical students	Brief Symptom Inventory, MPS – Hewitt, CIPS	Health professions students did not report significantly higher levels of perfectionism than other student populations; Self-oriented perfectionism positively and significantly correlated with distress; Strongest predictors of medical student distress were IP and socially-prescribed perfectionism

Ikbaal, M., et al.	2018	Malaysia	Quantitative, Cross-sectional; In-person questionnaires	256 4th year medical students	CIPS, RSES, PHQ9, Beck's Anxiety Inventory	Positive significant correlation between IP and anxiety and depression; Negative significant correlation between IP and self-esteem in medical students
LaDonna, K., et al.	2018	Canada	Qualitative	28 physicians	N/A	IP was perceived to occur at the extreme end of self-doubt in physicians. The culture of medicine may exacerbate or train feelings of inadequacy
Leach, P, et al.	2018	United States	Quantitative, Cross sectional	General surgeons and general surgery residents	CIPS, Single-item burnout measure	Surgical residents had higher CIPS scores than surgeons; Positive and statistically significant correlation between burnout and IP
Legassie, J., et al.	2008	Canada	Quantitative, Cross-sectional	48 internal medicine residents	CIPS, MBI	43.8% of residents had IP, 12.5% had burnout: No statistically significant correlation between IP and burnout
Mascarenhas, V, et al.	2018	India	Quantitative, Cross-sectional	150 medical interns	CIPS, RSES	44.7% and 41.3% of interns had moderate and high IP, respectively; Negative statistically significant association between IP and self-esteem
Oriel, K., et al.	2004	United States	Quantitative, Cross-sectional	194 family medicine residents	CIPS, RSES, State Anxiety Scale, Trait Anxiety Scale, DSM IV criteria	41% of women and 24% of men were impostors; Statistically significant positive correlation between impostor scores and depressive symptoms, Trait Anxiety scores, and State Anxiety scores; Statistically significant negative correlation between IP and self-esteem
Seeliger, et al.	2017	Germany	Quantitative, Cross-sectional	298 newly admitted first-year medical students	MPS-Hewitt, MPS-Frost, Big five inventory, General self-efficacy scale, PHQ-9, Generalized Anxiety Disorder-7	Medical students had lower scores for socially-prescribed perfectionism than the general population; Maladaptive perfectionism was the strongest predictor of depression and anxiety
Villwock, J., et al.	2016	United States	Quantitative, Cross-sectional	138 medical students	Young Impostor Scale, MBI – Human Services Survey	Female gender and fourth year of medical school were positively and significantly associated with IP; IP was positively and significantly associated with the burnout components of exhaustion, cynicism, emotional exhaustion, and depersonalization
Yu, J., et al.	2016	Korea	Quantitative, Cross-sectional	227 first-year and second-year premed medical students and first through fourth-year medical students	MPS – Hewitt, Self-efficacy scale by Kim and Park, MBI– student survey	Perfectionism showed a positive and statistically significant correlation with academic burnout, psychological distress, cynicism, and emotional exhaustion correlations and negatively correlated with self-confidence

Note: CBI = Copenhagen Burnout Inventory; CIPS = Clance's Impostor Phenomenon Scale; MPS = Multidimensional Perfectionism Scale; MBI = Maslach Burnout Inventory; RSES = Rosenburg Self-Esteem Scale.
